# Association of IL-6 -174G>C and -572G>C Polymorphisms with Susceptibility to Cervical Cancer and Ovarian Cancer

**DOI:** 10.31557/APJCP.2021.22.9.2867

**Published:** 2021-09

**Authors:** Ahmad Hashemzehi, Mojgan Karimi-Zarchi, Seyedeh Fatemeh Parsaeian, Fatemeh Asadian, Hossein Golestanpour, Sepideh Setayesh, Seyed Amir Shaker, Masoud Zare-Shehneh, Hossein Neamatzadeh

**Affiliations:** 1 *Department of Pharmaceutics, Faculty of Pharmacology, Tehran University of Medical Sciences, Tehran, Iran. *; 2 *Endometriosis Research Center, Iran University of Medical Sciences, Tehran, Iran. *; 3 *Department of Obstetrics and Gynecology, Firoozgar Hospital, Iran University of Medical Sciences, Tehran, Iran. *; 4 *Department of Obstetrics and Gynecology, Shahid Sadoughi University of Medical Sciences, Yazd, Iran. *; 5 *Department of Medical Laboratory Sciences, School of Paramedical Science, Shiraz University of Medical Sciences, Shiraz, Iran. *; 6 *Department of Genetics, Marvdasht Branch, Azad University, Marvdasht, Iran. *; 7 *Biotechnology Research Center, International Campus, Shahid Sadoughi University of Medical Sciences, Yazd, Iran.*; 8 *Student of Medicine, School of Medicine, Shiraz University of Medical Sciences, Shiraz, Iran. *; 9 *Department of Anatomy School of Medicine, Iran University of Medical Sciences, Tehran, Iran. *; 10 *Mother and Newborn Health Research Center, Shahid Sadoughi University of Medical Sciences, Yazd, Iran. *; 11 *Department of Medical Genetics, Shahid Sadoughi University of Medical Sciences, Yazd, Iran. *

**Keywords:** Cervical cancer, Ovarian Cancer, Interleukin 6, Polymorphism

## Abstract

**Background:**

During the past decades, the expansion of molecular development has had a key role in understanding the basis of gynecological cancer. Interleukin-6 (IL-6) is known to be involved in the pathogenesis of different cancers. Here, we evaluated the association of IL-6 -174G>C and -572 G>C polymorphisms with susceptibility to cervical and ovarian cancers in an Iranian population.

**Methods:**

A total of 131 cases with ovarian cancer, 124 cases with cervical cancer and 140 healthy subjects were enrolled to the study. DNA was extracted from peripheral blood cells of subjects to genotype the IL-6 -174G>C and -572 G>C polymorphisms by amplification refractory mutation system (RFLP) polymerase chain reaction (PCR).

**Results:**

There was a significant association of IL-6 -174G>C CC genotype (OR= 3.231, 95% CI: 1.130-9.239, p=0.029) and C allele (OR = 1.915; 95%CI: 1.266-2.896, p=0.002) with an increased risk of ovarian cancer. Moreover, the IL-6 -174G>C CC genotype (OR= 3.162, 95% CI: 1.094-9.141, p=0.034) and C allele (OR = 1.724; 95%CI: 1.129-2.633, p=0.012) was associated with increased risk of cervical cancer.

**Conclusions:**

This study showed that the IL-6 -174G>C polymorphism was associated with ovarian cancer and cervical cancer risk. However, IL-6 -572 G>C polymorphism was not associated.

## Introduction

The incidence of gynecological cancers, especially ovarian cancer and cervical cancer continues to increase in low and middle resource countries (Karimi Zarchi et al., 2014a; Karimi-Zarchi et al., 2014b). Ovarian cancer and cervical cancers are an important public health concern worldwide (He and Shen, 2017; Karimi-Zarchi, et al., 2020). Ovarian cancer is the fifth most common cancer and leading cause of death from gynecologic al cancer-related mortality in the Western world (Karimi-Zarchi et al., 2020). The most recent global statistic estimates 295,414 newly diagnosed cases of ovarian cancer every year and 184,799 annual deaths from this disease (Reid et al., 2017; Momenimovahed et al., 2019). Moreover, cervical cancer is the second most common cancer among women and fourth most frequently occurring gynecological cancer worldwide, with an estimated 528,000 new cases and 266,000 deaths among women each year (Acharya Pandey and Karmacharya, 2017; LaVigne et al., 2017). The etiology of ovarian cancer and cervical cancer is still not fully clarified, although a number of risk factors have been identified (Barbisan et al., 2012; Yu et al., 2013). Growing evidence demonstrates that genetic variant in cytokines and interleukins genes play a critical role in the development and prognosis of the ovarian cancer and cervical cancer (Singh et al., 2009).

Interleukin-6 (IL-6) is a multi-functional pro-inflammatory cytokine that has crucial roles in tumours progression through growth-promotion, anti-apoptotic activity, and modulation of immune function (Nogueira De Souza et al., 2006; Li et al., 2010). Moreover, IL-6 has homeostatic physiological roles besides its proinflammatory effects (Favalli, 2020). IL-6 does not only regulate tumor growth through direct effects on tumor cells but also indirectly via the tumor microenvironment, leading to induction of apoptosis, neovascularization and acute phase responses (Chonov et al., 2019). It is a major player in inflammation which accompanies or induces a wide range of disorders and/or malignancies such as traumatic brain injury, such as the development of atherosclerosis, ovarian steroid production, fertilization and implantation, coronary heart disease, osteoporosis, and allergic reactions (Chen et al., 2018). Thus, IL-6 is a strong candidate for mediating both local and systemic cancer-associated inflammatory responses.

The human IL-6 gene is mapped to chromosome 7p21-24 region, containing of 4 introns, with a 303 bp promoter and a total length of 5kb (Jafari-Nedooshan et al., 2019). It is well-known that the IL-6 polymorphisms are responsible for the regulation of the transcriptional activity during inflammation reaction (Garrote et al., 2005). To date, more than 50 single-nucleotide polymorphisms (SNPs) in promoter region of *IL-6* gene, which among these SNPs, two functional -174G>C and -572 G>C polymorphisms have been reported to affect the plasma levels of this cytokine (Chauhan and McGuire, 2008; Ren et al., 2016). A number of studies have shown that IL-6 -174G>C (rs1800795) and -572C>G (rs1800796) polymorphisms have a role in the predisposition to gynecological malignancies including cervical and ovarian cancers. However, these results are varied among different studies, partially caused by different designs, sample sizes and the diverse origins of selected populations. Moreover, considering that ovarian and cervical cancers are multifactorial diseases which are characterized by a disruption of the cytokines, we hypothesized that IL-6 -174G>C and -572 G>C polymorphisms might be associated with the risk these cancers.

## Materials and Methods

*Population*

All procedures in this study were in accordance with the ethical standards of the institutional or national research committee and with the 1964 Declaration of Helsinki and its later amendments or comparable ethical standards. The objective of the study was fully explained to all participants and a written informed consent was obtained from each participants. This study was approved by the Ethics Committee of the Azad University, Tehran. A total of 131 cases with ovarian cancer, 124 cases with cervical cancer and 140 healthy controls were recruited between May 2014 and January 2019.

*Genotyping*

The -174G>C and -572G>C variants at *IL-6* gene were systematically selected according to the previous studies in different populations. Peripheral blood samples were drawn in EDTA containing tubes from cases diagnosed with ovarian cancer, cervical cancer and cancer-free women selected as age-matched normal controls. A Commercially available kit for DNA isolation was used for DNA extraction, according to the manufacturer’s instructions kit (purchased from GeneAll Co., LTD). Primers were designed using Oligo software and NCBI BLAST search engine as presented in Table 1. Moreover, the SNP-ID, PCR product sizes and restriction enzyme used for each polymorphism are presented in Table 1. The PCR amplification was performed in a total volume of 20 µL reaction mixture, contain 4 µL genomic DNA, 10 µL Master mix 2x, 1 µL of each primers and 4 µL sterilized water. For -174G>C, the reaction mixtures were denatured at 94°C for 4 min, followed by 36 cycles of 94°C for 50 s, 64°C for 30 s, and 72°C for 1 min, with a final elongation at 72°C for 5 min. The PCR products were separated at 37°C overnight by 2% agarose gel electrophoresis and visualized under UV light. The genotypes were assessed as follows: a single 127-bp fragment represented the AA genotype; two fragments of 108 and 19 bp represented the CC genotype; and three fragments of 127, 108, and 19 bp represented the AC genotype. For -572G>C, the reaction mixture was subjected to 35 cycles of 45 seconds at 94°C, 60 seconds at 65°C, and 25 seconds at 72°C. The final cycle was 10 minutes at 72°C. The PCR products were digested with NlaIII and MbiI at 37°C overnight for -174G>C and -572G>C variants, respectively. Then, the products were electrophoresed on 2.0% DNA agarose gel and stained with ethidium bromide and visualized using a gel documentation system.

*Statistical Analysis*

Genotype frequencies were estimated for both patients with ovarian cancer and cervical cancer as well as for healthy subjects. Hardy-Weinberg equilibrium models in controls were determined for IL-6 -174G>C and -572G>C polymorphisms using a Chi-square test. Comparisons between genotype distribution and association with selected clinical data were performed with the Chi-square test and Fisher’s exact test. The risk for genotypes and alleles for both IL-6 polymorphisms was determined as odds ratios (ORs) and 95% confidence intervals (CIs). All of the statistical analyses were performed in SPSS version 20.0 (SPSS, Chicago, IL) and statistical significance was set at two-sided P ≤ 0.05.

## Results

Screening for the IL-6 -174G>C and -572G>C polymorphisms in ovarian cancer and cervical cancer was performed in this study. The distribution of genotypes for IL-6 -174G>C and -572G>C polymorphisms was in agreement with the Hardy-Weinberg equilibrium (p=0.523 and p=0.951) in the control subjects.

Genotypes and alleles frequency of for IL-6 -174 G>C and -572G>C variants are listed in Table 2 and 3. As shown in Table 3, there was a significant differences between ovarian cancer and cervical cancer patients and controls for IL-6 -174 G>C variant. Statistically significant association of the IL-6 -174G>C CC genotype (OR= 3.231, 95% CI: 1.130-9.239, p=0.029) and C allele (OR = 1.915; 95%CI: 1.266-2.896, p=0.002) with the increased risk of ovarian cancer was found. Moreover, statistically significant association of the IL-6 -174G>C CC genotype (OR= 3.162, 95% CI: 1.094-9.141, p=0.034) and C allele (OR = 1.724; 95%CI: 1.129-2.633, p=0.012) with the increased risk of ovarian cancer was found. However, there were no significant differences between ovarian cancer and cervical cancer patients and controls for IL-6 -572G>C variant. 

**Table 1:** Genotyping Features and Sequences of PCR Amplification Primers for Genotyping the IL-6 Polymorphisms

Polymorphism	SNP-ID	Position	Sequence	Annealing	RE	Genotype
IL-6 -174 G>C	rs1800795	chr7:22727026	F: 5'-TTGTCAAGACATGCCAAGTGCT-3'	57 °C	NlaIII	G:244, 133, 11
			R: 5'-GCCTCAGAGACATCTCCAGTCC-3'			C: 133,111, 56
IL-6 -572 G>C	rs1800796	chr7:22726627	F: 5'-GGAGACGCCTTGAAGTAACTGC-3'	55 °C	MbiI	G:102, 61
			R: 5'-GAGTTTCCTCTGACTCCATCGCAG-3'			C: 163

**Table 2:** Genotype Frequencies of the IL-6 Polymorphisms in Cases with Ovarian Cancer and Controls

Polymorphism	Cases (n=131)	Controls (n=140)	Odds Ratio		
			OR	90% CI	P-Value
IL-6 -174 G>C					
Genotypes					
GG	72 (54.9%)	98 (70.0%)	Ref.		
GC	45 (34.4%)	37 (26.4%)	1.457	0.865-2.452	0.157
CC	14 (10.7%)	5 (3.6%)	3.231	1.130-9.239	0.029
Alleles					
G	189 (72.1%)	233 (83.2%)	Ref.		
C	73 (27.9%)	47 (16.8%)	1.915	1.266-2.896	0.002
IL-6 -572G>C					
Genotypes					
GG	69 (52.7%)	72 (51.4%)	Ref.		
GC	54 (41.2%)	57 (40.8%)	1.021	0.629-1.658	0.932
CC	8 (6.1%)	11 (7.8%)	0.763	0.297-1.960	0.574
Alleles					
G	192 (73.3%)	201 (71.8%)	Ref.		
C	70 (26.7%)	79 (28.2%)	0.928	0.636-1.353	0.697

OR, Odds Ratio; CI, Confidence Interval.

**Table 3:** Genotype Frequencies of the IL-6 Polymorphisms in Cases with Cervical Cancer and Controls

Polymorphism	Cases (n=124)	Controls (n=140)	Odds Ratio		
			OR	90% CI	P-Value
IL-6 -174 G>C					
Genotypes					
GG	73 (58.9%)	98 (70.0%)	Ref.		
GC	38 (30.6%)	37 (26.4%)	1.23	0.720-2.102	0.449
CC	13 (10.5%)	5 (3.6%)	3.162	1.094-9.141	0.034
Alleles					
G	184 (74.2%)	233 (83.2%)	Ref.		
C	64 (25.8%)	47 (16.8%)	1.724	1.129-2.633	0.012
IL-6 -572G>C					
Genotypes					
GG	61 (49.2%)	72 (51.4%)	Ref.		
GC	51 (41.1%)	57 (40.8%)	1.017	0.622-1.663	0.945
CC	12 (9.7%)	11 (7.8%)	1.256	0.534-2.959	0.601
Alleles					
G	173 (69.8%)	201 (71.8%)	Ref.		
C	75 (30.2%)	79 (28.2%)	1.103	0.758-1.606	0.609

OR, Odds Ratio; CI, Confidence Interval.

## Discussion

Genetic variants at the promoter region of the *IL-6 *gene can result in variations in transcription and influence the susceptibility to various cancers. The importance of studying the genetic variants at *IL-6 *gene in different populations is underlined by the fact that there are important ethnic differences. However, a few studies have been conducted to determine the association of IL-6 polymorphisms with ovarian cancer and cervical cancer risk. Thus, in this study the association of IL-6 -174G>C and -572G>C polymorphisms with ovarian cancer and cervical cancer in Iranian women was evaluated.

Our results showed that IL-6 -174G>C is significantly associated with increased risk of ovarian cancer in the Iranian population. However, no significant association was found between IL-6 -572G>C polymorphism and ovarian cancer risk. Bushley et al., (2004) in study have evaluated the association of IL-1α, IL-β, IL-6, IL-10, and IL-18 polymorphisms with risk of epithelial ovarian cancer. However, their results failed to support an association between selected polymorphisms at IL-1α, IL-β, IL-6, IL-10, or *IL-18* gene and increased risk of ovarian cancer in USA. Lu et al., (2016) have examined the association of genetic variants at inflammatory response genes such as PPARG Pro12Ala, IL6-174G/C, E-selectin S128R, NFKB1-94 ins/del, NFKBIA-826C/T, and ICAM-1 K469E with susceptibility to ovarian cancer in a Chinese population. Their results also did not found a significant association. However, Garg et al., (2006) have indicated that homozygote wild genotype (GG) of IL-6 -174 G>C polymorphism has a strong, independent, and favorable impact on survival in women with ovarian cancer and peritoneal carcinoma (Garg et al., 2006). Similarly, pooled data in a meta-analysis supported that recessive genetic model (GG vs. GC+CC) of IL-6 -174 G>C polymorphism is significantly associated with increased survival of women with ovarian cancer, peritoneal cancer and other malignancies. On the other hand, their pooled results showed a protective effect for GG genotype of IL-6 -174 G>C polymorphism on development of ovarian cancer {Formatting Citation}.

The current study showed that the IL-6 -572G>C polymorphism with risk of cervical cancer. The previous studies on the association of IL-6 -174G>C and -572G>C polymorphisms with cervical cancer risk reported inconsistent results. Pu et al., in case-control among 360 cervical cancer patients and 728 controls revealed that IL-6 rs1800795 and rs2069837 variants were associated with increased cervical cancer risk in chinese women (Pu et al., 2016). Zidi et al., (2017) also in a case-control showed that IL-6 rs1800795 and rs1474348 polymorphisms are major risk factors of cervical cancer among Tunisian women. Duan et al., (2018) in a meta-analysis of seven article evaluated the association of IL-6 -174G>C (rs1800795) polymorphism with cervical cancer. They reported that the IL-6 -174G>C polymorphism was a low-penetrance susceptibility variant for cervical cancer. However, de Moura et al., (2020) in a meta-analysis on 7 cytokine genes showed that 10 SNPs in cytokine genes including IL-6 (rs1800795) were associated with increased risk for cervical cancer. Karimi et al., (2020) in other meta-analysis showed that Pooled ORs revealed that the IL-6 rs1800795 polymorphism was significantly associated with an increased risk of cervical cancer, especially in Asian women.

In summary, this study showed that IL-6 -174G>C polymorphism was associated with susceptibility to ovarian cancer and cervical cancer. However, we did not find a significant association between IL-6 -572G>C polymorphism and ovarian cancer and cervical cancer in our population. Thus, the IL-6 -174G>C polymorphism is a potential to be evaluated as prognostic biomarkers predicting or identifying cases of high risk of ovarian cancer and cervical cancer. However, all cancer cases and controls were selected from two hospitals and, therefore may not ideally represent the general population. 

## Author Contribution Statement

Conceived and designed the study and experiments: AH, MKZ, SS. Performed the experiments: LZ and MZS

Analyzed the data: SAD, HN and FA. Contributed reagents/materials/analysis tools: HN and SKMZS. Wrote the paper: AS, SAD and LZ. All authors reviewed the manuscript.
